# Psoas Muscle Index and Density as Prognostic Predictors in Patients Hospitalized with Acute Pancreatitis

**DOI:** 10.3390/jcm13216314

**Published:** 2024-10-22

**Authors:** Alena Kirzhner, Anton Rossels, Danielle Sapojnik, Hilla Zaharoni, Ramon Cohen, Guy Lin, Tal Schiller

**Affiliations:** 1 Department of Internal Medicine A, Kaplan Medical Center and Faculty of Medicine, Hebrew University of Jerusalem, Jerusalem 9190401, Israel; 2 Department of Radiology, Kaplan Medical Center, Faculty of Medicine, Hebrew University of Jerusalem, Jerusalem 9190401, Israel; 3 Department of Clinical Nutrition, Kaplan Medical Center, Faculty of Medicine, Hebrew University of Jerusalem, Jerusalem 9190401, Israel; 4 Department of Diabetes, Endocrinology and Metabolism, Kaplan Medical Center, Faculty of Medicine, Hebrew University of Jerusalem, Jerusalem 9190401, Israel; talsc1@clalit.org.il; 5 Department of Internal Medicine B, Kaplan Medical Center, Faculty of Medicine, Hebrew University of Jerusalem, Jerusalem 9190401, Israel; 6 Department of General Surgery B, Kaplan Medical Center, Faculty of Medicine, Hebrew University of Jerusalem, Jerusalem 9190401, Israel

**Keywords:** acute pancreatitis, psoas muscle area, psoas muscle index, psoas muscle density, sarcopenia

## Abstract

**Background**: Early prognostication of acute pancreatitis (AP) patients for those at high risk of complications during hospitalization can facilitate clinical decision-making. Sarcopenia has been proven to be a risk factor for poor prognosis in patients with AP. We aimed to evaluate the association between the muscle parameters measured in computed tomography (CT) and the clinical outcomes of hospitalized patients with AP. **Methods**: A total of 132 consecutive patients hospitalized between 1 January 2015 and 31 December 2021 for AP with a valid CT scan were analyzed. The first CT conducted during hospitalization was analyzed for psoas muscle area (PMA), index (PMI), and density (PMD) at the L3 vertebral level. The main adverse outcomes indicating a worse prognosis were the development of extrapancreatic complications, infections, ICU transfer, in-hospital mortality, and hospitalization length. **Results**: The lowest tertile of PMI, as a surrogate for sarcopenia, was significantly correlated with increased rates of extrapancreatic complications, infections, and longer hospitalizations. It was additionally correlated with a worse CT severity index. The results for PMA and PMD also showed worse outcomes, largely mirroring the results for PMI. Although in-hospital mortality was relatively low, none of the patients died in the highest tertile of PMI. A clear cutoff with sufficient predictive capability could not be found. **Conclusions**: A low psoas muscle index can serve as an additional potential predictive marker for more severe disease and worse outcomes in hospitalized acute pancreatitis patients. More studies are needed to determine its combination with existing prediction tools.

## 1. Introduction

Acute pancreatitis (AP) refers to an inflammatory condition of the pancreas. The reported annual incidence of AP ranges from 4.9 to 35 per 100,000 people; however, the incidence is rising due to increased rates of obesity and gallstones [1,2,3]. AP is the third leading gastrointestinal cause of hospitalization in the United States [4]. Gallstones and chronic alcohol use disorder are the leading causes of AP [5].

Approximately 15 to 25 percent of all patients with AP develop moderately severe (MSAP) or severe AP (SAP) [5]. While mortality rates of mild AP range between 3 and 10%, they increase dramatically to 36 to 50% in SAP [4]. Severity prediction may help identify patients at increased risk for morbidity and mortality, assisting in early triaging to intensive care units (ICUs) and selecting patients for specific interventions. Necrotizing pancreatitis represents the most severe form of parenchymal injury in AP and occurs in 5–10% of patients [6]. MSAP and SAP can manifest multiple infectious or noninfectious extrapancreatic complications [7].

Currently, disease occurrence and mortality are predicted by the combined use of clinical data, biochemical analysis, and imaging; however, approximately 20–30% of SAP cases are misclassified as having a milder disease based on clinical data [8].

Multiple scoring systems, such as the Atlanta classification, the Ranson score, the Glasgow score, the Acute Physiology and Chronic Health Examination (APACHE), the Japanese severity score (JSS), the Harmless Acute Pancreatitis score (HAPS), and the Bedside Index for Severity in Acute Pancreatitis (BISAP) score, have been developed to predict disease severity and outcome [9]. Some can be performed on admission, while others can only be obtained after the first 48 to 72 h or later. However, given their low specificity and complexity of use, the American Gastroenterological Association Guidelines of 2018 recommend a global approach, including age, nutritional status, an assessment of comorbidities, and the presence of hemodynamic, biological, or radiological disorders for the early recognition of SAP rather than the use of these scores [10]. Thus, there is a need for new tools and predictive models for a more accurate prognostication of SAP at an early stage with high sensitivity and specificity.

Sarcopenia has been recognized as a potential risk factor for adverse outcomes in AP. Sarcopenia is defined as a progressive and generalized skeletal muscle disorder characterized by loss of both skeletal muscle mass and strength, which is proved to be an independent poor prognostic factor compared to weight loss and BMI. This was demonstrated in various settings, such as increased in-hospital mortality in acute necrotizing pancreatitis [11] and reduced survival in malignant diseases and is a significant predictor of operative complications following pancreatectomy [12,13,14]. Sarcopenic obesity has recently been shown to be a significant risk factor for postoperative morbidity after pancreatic surgery [15].

Contrast-enhanced computed tomography (CT) is an important tool for assessing and monitoring AP. Previous studies have demonstrated that CT can be used to estimate muscle mass. Utilizing CT for this purpose is simple and feasible compared to other methods for assessing body composition, such as dual X-ray absorptiometry, which is not available in the acute setting, or bioimpedance methods that are influenced by hydration status. CT-measured psoas muscle thickness normalized to height was shown to be a promising predictor of morbidity and mortality in several chronic diseases, such as in patients undergoing hemodialysis, chronic liver disease, and cardiovascular and oncologic disease [16,17,18].

Several CT-measured sarcopenic indices can be used for this assessment. Quantifying the psoas muscle area (PMA) at the third lumbar vertebra level was shown to correlate well with whole-body skeletal muscle mass. When adjusted for height, it is called the psoas muscle index (PMI), calculated as PMA/height^2^ (cm^2^/m^2^). Psoas muscle density (PMD) has received growing recognition as a predictor of muscle quality as a surrogate for myosteatosis, i.e., fat-infiltrating muscle. However, no clear cutoff exists for assessing reduced skeletal muscle mass and PMD. Various cutoffs may be associated with race, age, comorbidities, anthropometric data, or method of determining cutoff value.

The prognostic implications of CT sarcopenic indices in AP have been explored in a few studies [19,20,21,22]. Three were conducted on Asian populations [19,20,21] and one was conducted on AP patients admitted to ICUs with organ failure [22].

We aimed to evaluate the association between muscle parameters as measured in CT and adverse outcomes for hospitalized patients with AP.

## 2. Materials and Methods

### 2.1. Study Design and Definitions

In total, 327 consecutive patients older than 18 years were admitted to the Department of General Surgery B in Kaplan Medical Center with a principal diagnosis of AP from January 2015 to December 2021. Among the subjects, 132 cases had complete abdominal and pelvic CT imaging records and were retrospectively reviewed. CT scans were reviewed if performed up to a week from the hospitalization date. The diagnosis of AP at discharge was identified by the International Classification of Diseases at discharge, Tenth Revision (ICD-10). This study was approved by the Institutional Review Board (IRB) of Kaplan Medical Center (KMC-000-9-2022), date of approval 10 March 2022. The first hospitalization with AP was considered the index hospitalization.

All reviewed cases were validated if patients met at least two of the following three manifestations: acute-onset characteristic upper abdominal pain; elevated levels of pancreatic enzymes (serum amylase or lipase three times above the upper limit of normal levels); and findings of pancreatic inflammation in CT suggesting AP [23].

The exclusion criteria comprised the following: 1. chronic diseases that can affect test result accuracy, such as background malignant disease or known skeletal muscle disease; 2. AP caused by periampullary tumor, pre-malignant, and malignant conditions 3. patients with known chronic pancreatitis or signs of chronic pancreatitis in CT scans; 4. expected life expectancy of less than six months for any reason; 5. patients with missing medical record data; 6. pregnant patients; and 7. insufficient CT quality or if the CT was performed more than seven days after hospitalization.

The etiology of AP was classified as related to gallstones, alcohol, hypertriglyceridemia, and other (post-endoscopic procedures, traumatic). If no etiology could be identified based on the medical records, it was classified as unknown.

The Ranson score was calculated to predict the clinical severity of AP with parameters collected between 24 and 48 h after admission [9].

Infectious complications included any of the following diagnoses: sepsis; septicemia; septic shock; abdominal infection—cholangitis or peritonitis; pneumonia; and necrotizing pancreatitis.

Nutritional assessments were performed according to the Nutrition Risk Assessment (NRA) score between 24 and 72 h after hospitalization. The NRA involves BMI or weight loss before hospitalization, nutritional impairments (calories and liquid consumed, ability to eat), the patient’s comorbidities, the number of chronic medications, physical and mental functioning (bedridden, chewing or swallowing problems, communication needs, etc.), albumin level (or other related laboratory abnormalities), and presence of pressure ulcers [24]. If the NRA score was more than 7, the patient was defined as having “severe nutritional risk”, an NRA score of 3 to 7 was defined as “moderate nutritional risk”, and an NRA score of 0–2 was defined as “mild nutritional risk”.

### 2.2. Data Collection

Detailed clinical information was extracted from hospital electronic medical records (EMRs). The variables included both demographic and anthropometric information, smoking status (current or past smoking), alcohol consumption, and comorbidities; data on AP—etiology and complications; laboratory blood tests at admission and during hospitalization; contrast-enhanced abdominal CT; length of hospitalization (LOH); and in-hospital mortality data.

**Figure 1:**
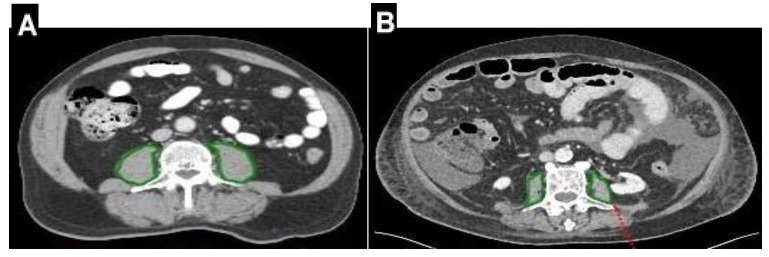
Axial CT images at the L3 vertebral level. The green line shows the outline of the psoas muscle. Measurements were performed in a semi-automated fashion with manual outlining of psoas muscle borders. Two pictures of axial CT images in two different patients are shown: (**A**) a patient with mild pancreatitis and PMA 28 cm^2^ and (**B**) a patient with PMA 11 cm^2^ and marked ascites.

The first CT performed after the onset of AP was retrieved for all included patients. The CT images were retrospectively analyzed manually by a trained radiologist (AR). The data extracted included the following: 1. PMA at the middle of the third lumbar vertebra level (L3) left and right, measured in two consecutive images as a measure of whole-body skeletal muscle mass; 2. PMI, calculated using the equation: PMA (cm^2^)/height^2^ (m^2^); and 3. PMD is measured in Hounsfield units (HU). Only CTs performed in the native or venous phases were used. All muscle parameters were measured using Workstation software (Phillips IntelliSpace Portal v6.0.2.32500) (Figure 1).

As no clear cutoff exists for the diagnosis of sarcopenia, PMA and PMI were further divided into tertiles, with the highest tertile serving as a reference (the least amount of sarcopenia). Similarly, PMD was categorized into tertiles of <39 HU, 39–45 HU, and >45 HU, with the highest HU serving as a reference.

The CT severity index of AP was assessed using the Balthazar grade, as the most used score to evaluate AP severity and the presence and degree of pancreatic inflammation and necrosis [9]. The assessment was divided into no AP per CT, mild, moderate, and severe.

Extrapancreatic complications on CT included acute pleural effusion; ascites; vascular complications (splenic and portal vein thrombosis, hemorrhage); gastrointestinal bleeding; and perforation.

## 3. Statistical Analyses

Categorical and nominal variables were reported as frequencies and percentages. Continuous variables were assessed for normality using the Shapiro–Wilk test. Comparisons between continuous variables were performed using the independent sample t-test for normally distributed data and the Mann–Whitney U test for non-normally distributed data. Associations between categorical and nominal variables were analyzed using Pearson’s chi-square (χ^2^) test or Fisher’s exact test, as appropriate. To find a cutoff for PMI and PMD, a receiver operating curve (ROC) was implemented. The ROC is a graphical representation of the diagnostic accuracy of the test, with the curve demonstrating the trade-off between sensitivity and specificity. The closer the curve follows the upper-left border of the ROC space, the more accurate the test.

Statistical significance was set at a two-tailed *p*-value of <0.05. All statistical analyses were conducted using IBM SPSS Statistics for Windows, version 29.0.

## 4. Results

### 4.1. Patient Characteristics

Out of 327 eligible AP patients, 195 patients (60%) were excluded because CT was either not performed (75%), was of insufficient quality (4%), was performed after more than a week (15%), or the patient had signs of chronic pancreatitis (6%). The final study cohort consisted of 132 patients, of whom 64% were males with a mean age of 59 ± 13 and a BMI of 28.4 ± 5.6 kg/m^2^. Among 45% of the patients, no distinct etiology could be identified from the EMR, 37% had gallstones, 7% had hypertriglyceridemia, and 5% had excessive alcohol consumption. A third were obese and a third had diabetes (Table 1).

The Ranson score was calculated for 88 patients (67%), and over half met the criteria for SAP. When assessing severity according to CT, only 14 (11%) met the criteria for SAP.

PMI, PMA, and PMD were significantly lower in patients older than 60 years compared to those younger than 60 years (4.92 ± 1.73 cm^2^/m^2^ vs. 6.01 ± 1.89 cm^2^/m^2^; 13.9 ± 5.4 cm^2^ vs. 17.7 ± 6.6 cm^2^; 39 ± 8 HU vs. 45 ± 6, HU, *p* < 0.001, respectively, for all parameters).

The PMA and PMI were significantly higher in males compared to females (18.6 ± 5.5 cm^2^ vs. 10.4 ± 3.6 cm^2^ and 6.18 ± 1.17 cm^2^/m^2^ vs. 4.06 ± 1.41 cm^2^/m^2^, respectively, *p* < 0.001). The PMD values were comparable between males and females (43 ± 7 HU vs. 40 ± 8 HU, respectively, *p* = 0.082).

### 4.2. Associations of PMI and PMD with Severity of Acute Pancreatitis

As demonstrated in Table 2, the patients were divided into tertiles both for PMI and PMD (the highest tertile represents the least amount of sarcopenia and serves as a reference). Patients with reduced PMI were older (63 ± 13.0 vs. 55.5 ± 13.3, *p* = 0.01) and had a significantly higher rate of extrapancreatic complications (68% vs. 14%, *p* = 0.046), severe pancreatitis in CT images (24% vs. 3%, *p* = 0.006), and more infections (34% vs. 7%, *p* = 0.003) than patients with a higher PMI. The in-hospital mortality rate was 5% of the entire cohort, and none of this came from the highest PMI tertile. LOH was also significantly higher in patients with the lowest PMI than in those with the highest. The analysis results for PMA and PMD largely mirrored those of PMI, with the lowest tertiles consistently showing worse outcomes than the highest (Table 2 and Supplementary Table S1).

ROC analysis was performed to determine a cutoff for each of the parameters and analyze their predictive capacity. Regrettably, all areas under the curve were small and no cutoff values for severe AP or adverse outcomes could be retrieved.

## 5. Discussion

The results of the current study demonstrate that patients in the lowest tertile of PMI had more severe outcomes of AP than those in the highest tertile. Patients with a lower PMI had worse clinical outcomes with more infections and higher rates of extrapancreatic complications, and this translated to significantly longer hospitalizations. A lower PMI correlated with a worse severity index in CT images. The results of PMD mirrored those of PMI and correlated with Ranson’s score severity index. Although in-hospital mortality rates were low, none happened in the highest PMI group.

Our results are mostly in line with several existing studies examining the CT parameters of sarcopenia as predictors of AP outcomes, albeit in different populations and with different chosen outcomes. In a study by Farquhar et al. [22], SAP patients admitted to an ICU with organ failure were assessed for mortality at 30 days, 3 months, and 12 months. Among 141 patients, sarcopenia and sarcopenic obesity were highly prevalent, comprising 79% and almost half of patients at admission, respectively. The patients’ characteristics were similar to those of our cohort. PMI cutoffs were based on sex and BMI. The results of the study demonstrate that sarcopenic obesity was a significant predictor of mortality on admission. On multivariate analysis, the presence of sarcopenic obesity, advanced age, and multiorgan failure were independent predictors of overall mortality in severe AP [22].

Several studies conducted on Asian populations also showed correlations between muscle parameters and AP outcomes. In a study from China, Fu et al. [19] found that among 269 patients with AP, PMA served as a good predictor of severity and complications, determined according to the revised Atlanta criteria. In another study by Zhou et al. [20], assessing fat and muscle cross-sectional areas at the level of the L3 vertebra body in 392 patients with AP, it was found that body composition might be key to predicting clinical outcomes of patients with AP. CT-assessed visceral adipose tissue (VAT) and skeletal muscle attenuation (SMA) were significantly associated with the development of SAP. SMA was associated with mortality rates in patients with SAP. The reported mortality rate was 5.1%, similar to the reported rate in our study. Notably, a visceral adipose tissue loss of ≥17% within 1 year was a protective factor for recurrent AP [20]. A third Korean study investigated both fat and muscle areas (skeletal muscle area was measured, including psoas, paraspinal, and abdominal wall muscles and excluding intra-abdominal visceral muscles) from abdominal CT scans at the L3 vertebral levels of patients with AP and suggested that a combined approach, both fat and muscle distribution, can be used for predicting the severity of AP [21]. The prevalence of various local complications and persistent organ failure was increased in patients with a higher visceral fat-to-muscle ratio [21]. A study that included 507 patients with necrotizing pancreatitis showed that lower skeletal muscle density was associated with increased disease severity [25]. However, in a study from Turkey on 127 newly diagnosed AP patients, skeletal muscle parameters, PMI, and PMD were not predictors of clinical course [26].

O’Leary et al. showed that the estimation of abdominal fat distribution parameters from CT scans performed on patients with AP indicates a strong association between visceral fat, severe AP, and the subsequent development of systemic complications [27].

Taken together, most studies show worse clinical outcomes, whether during hospitalization or post-hospitalization, with lower PMI and PMD. Given that muscle mass may deplete further during prolonged hospitalization, this in turn may prolong recovery [19,22]. This makes nutritional evaluation and support during hospitalization of paramount importance. In our cohort, only 38% of patients underwent nutritional assessment during hospitalization. Nutritional education may be continued after discharge, and further studies are needed to determine whether these interventional approaches benefit body composition and long-term prognosis.

To date, there is no clear cutoff for PMI and PMD below which risk increases. Cutoffs are dependent on sex, age, BMI, ethnicity, and other parameters. We therefore used tertiles and not an arbitrary cutoff for risk determination. ROC analysis for the entire cohort was performed, but, regrettably, all areas under the curve were small, and no cutoff values for severe AP or clinical outcome could be retrieved.

A study from China conducted by Fu et al. [19] determined the CT-assessed cutoff value of reduced muscle mass in patients with AP and assessed the effect of reduced muscle mass on the severity and early complications of AP. The study showed that PMA cutoff values of 11.50 cm^2^ for men and 8.22 cm^2^ for women can be considered cutoff values for reduced muscle mass. AP patients with PMAs below the cutoff values had higher rates of complication and organ failure than those with PMAs above the cutoff. Furthermore, PMA was independently associated with the severity of AP [19]. The cutoff values in the present study were determined for an Asian population and could be significantly lower than those observed in a European population.

The strengths of our study include a well-characterized cohort with multiple endpoints reflecting the severity of AP. Several limitations do exist, including a relatively small cohort, the fact that CT scans were performed up to a week post-admission, which might have influenced measurements, and selection bias, as CT was not routinely performed, meaning only selected patients with a more severe clinical course, deterioration, or lack of sufficient improvement were referred for CT.

## 6. Conclusions

In conclusion, CT-based parameters for sarcopenia assessment, such as psoas muscle index and psoas muscle density, have emerged as potential additional tools in evaluating the prognosis of patients with acute pancreatitis. Our findings support the available evidence that lower indices are associated with a higher complication rate in hospitalized patients with acute pancreatitis and highlight the importance of considering muscle mass and quality in the overall assessment and management of acute pancreatitis. These patients can further benefit from early nutritional support and guidance in the hospital and thereafter. Future prospective studies should investigate whether routine assessment of these parameters could be of added value in current CT severity index models, in predicting patient outcomes, and in determining whether treatment allows for counteracting the deleterious effects of body composition alterations.

## Figures and Tables

**Table 1:** Characteristics of patients hospitalized with acute pancreatitis.

Characteristic	All Patients (*n* = 132)
Age, years, mean ± SD	59 ± 13
Male, *n* (%)	84 (64)
BMI, mean ± SD	28.4 ± 5.6
Smoking, *n* (%)	44 (33)
Etiology of AP, *n* (%):	
Unknown	59 (45)
Biliary	49 (37)
Hypertriglyceridemia	9 (7)
Alcohol consumption	7 (5)
Other	8 (6)
Comorbidities, *n* (%):	
Hypertension	71 (54)
Hyperlipidemia	69 (53)
Diabetes	50 (38)
Obesity	47 (36)
Ischemic heart disease	25 (19)
Metabolic-associated fatty liver disease	10 (8)
Renal failure	9 (7)
Ranson criteria *:	
Non-severe, *n* (%)	37 (42)
Severe, *n* (%)	51 (58)
Ranson score, mean ± SD	3 ± 2
Nutritional status, *n* (%):	
NRA > 8	31 (62)
NRA 3–7	17 (34)
NRA 0–2	2 (4)
Nutritional consultation in hospital, *n* (%)	50 (38)
CT severity index of AP, *n* (%):	
No AP	15 (11)
Mild	79 (60)
Moderate	24 (18)
Severe	14 (11)
Data of CT, mean ± SD:	
Timing of CT, days	3 ± 3
PMA, cm^2^	15.6 ± 6.3
PMD, HU	42 ± 8
PMI, cm^2^/m^2^	5.42 ± 1.88
Outcomes, *n* (%):	
Extrapancreatic complications	72 (55)
Infection	24 (18)
Transfer to ICU	8 (6)
In-hospital mortality	7 (5)
LOH (days), mean ± SD	6 (7)

SD: standard deviation; BMI: body mass index; AP: acute pancreatitis; NRA: National Risk Assessment; CRP: C-reactive protein; CT: computed tomography; PMA: psoas muscle area; PMI: psoas muscle index; PMD: psoas muscle density; HU: Hounsfield units; ICU: intensive care unit; LOH: length of hospitalization. * Available for 88 patients.

**Table 2:** Association between psoas muscle index, psoas muscle density parameters, and severity of acute pancreatitis.

Characteristic	PMI < 4.35 cm^2^/m^2^ (*n* = 44)	PMI = 4.35–6.29 cm^2^/m^2^ (*n* = 44)	PMI > 6.29 cm^2^/m^2^ (*n* = 43)	*p* Value	PMD < 39 HU (*n* = 46)	PMD = 39–45 HU (*n* = 44)	PMD > 45 HU (*n* = 42)	*p* Value
Age, years, mean ± SD	63.2 ± 13.0	58.7 ± 12.6	55.5 ± 13.3	0.010	65.0 ± 11.0	57.0 ± 13.6	54.7 ± 13.0	<0.001
BMI, mean ± SD	28.5 ± 7.5	27.8 ± 4.4	29.0 ± 4.2	0.471	29.9 ± 6.6	27.9 ± 5.4	27.4 ± 4.1	0.106
Ranson criteria’s, *n* (%) *:								
Non-severe	13 (30)	12 (27)	12 (28)	0.823	10 (29)	15 (50)	12 (52)	0.113
Severe	21 (48)	16 (36)	14 (33)		25 (71)	15 (50)	11 (48)	
Ranson score, mean ± SD	2.9 ± 1.5	2.6 ± 1.9	2.4 ± 1.4	0.571	3.2 ± 1.7	2.2 ± 1.5	2.3 ± 1.5	0.038
High-sensitivity CRP first, mg/dL, mean ± SD	44.9 ± 85.0	45.6 ± 62.4	41.1 ± 83.7	0.794	38.1 ± 57.8	47.8 ± 87.9	44.0 ± 80.4	0.378
High-sensitivity CRP second, mg/dL, mean ± SD	25.7 ± 40.8	24.2 ± 40.5	81.9 ± 130.3	0.587	40.8 ± 52.0	23.1 ± 48.2	43.9 ± 98.7	0.076
CT severity index of AP, *n* (%):								
Mild	18 (49)	28 (68)	33 (87)	0.006	23 (55)	26 (72)	30 (77)	0.160
Moderate	10 (27)	9 (22)	4 (11)		11 (26)	8 (22)	5 (13)	
Severe	9 (24)	4 (10)	1 (3)		8 (19)	2 (6)	4 (10)	
Extrapancreatic complications, *n* (%)	30 (68)	23 (52)	18 (14)	0.046	29 (63)	24 (55)	19 (45)	0.246
Infection, *n* (%)	15 (34)	6 (14)	3 (7)	0.003	10 (22)	10 (23)	4 (10)	0.210
Transfer to ICU, *n* (%)	5 (11)	2 (5)	1 (2)	0.185	5 (11)	1 (2)	2 (5)	0.212
In-hospital mortality, *n* (%)	3 (7)	4 (9)	0 (0)	0.209	3 (7)	2 (5)	2 (5)	0.900
LOH (days), mean ± SD	9 ± 10	6 ± 7	4 ± 3	0.010	8 ± 9	6 ± 4	6 ± 8	0.044

PMI: psoas muscle index; PMD: psoas muscle density; SD: standard deviation; BMI: body mass index; CRP: C-reactive protein; CT: computed tomography; AP: acute pancreatitis; ICU: intensive care unit; LOH: length of hospitalization. * Available for 88 patients.

## Data Availability

The data presented in this study are available on request from the corresponding author.

## Supplementary Materials

The following supporting information can be downloaded at https://www.mdpi.com/article/10.3390/jcm13216314/s1, Supplementary Table S1. Association between psoas muscle area parameters and severity of acute pancreatitis.
