# Supplementary material for: Psoas Muscle Index and Density as Prognostic Predictors in Patients Hospitalized with Acute Pancreatitis
Source: J Clin Med. 2024 Oct 22;13(21):6314. doi: 10.3390/jcm13216314 (PMC11547049; doi:10.3390/jcm13216314)
Supplement: Supplementary file 1 [file jcm-13-06314-s001.zip › jcm-3236170-supplementary.pdf]

Supplementary Table S1. Association between psoas muscle area parameters and severity of acute pancreatitis.

Characteristic	PMA<12cm^2 (N=40)	PMA=12-18cm^2 (N=52)	PMA>18cm^2 (N=40)	P value
Age, years, mean ± SD	63.8 (12.7)	58.8 (13.1)	54.7 (12.7)	**0.003**
BMI, mean ± SD	28.9 (7.9)	27.6 (4.2)	29.1 (4.2)	0.428
Ranson criteria's, n (%)*:				
Non-severe	11 (37)	15 (44)	11 (46)	0.757
Severe	19 (63)	19 (56)	13 (54)	
Ranson score, mean±SD	2.8 (1.5)	2.6 (1.8)	2.5 (1.6)	0.774
High-sensitive CRP first, mg/dL, mean±SD	31.0 (68.6)	62.3 (79.9)	40.0 (80.5)	0.268
High-sensitive CRP second, mg/dL, mean±SD	22.1 (36.1)	30.3 (49.0)	73.9 (122.7)	0.443
CT severity index of AP, n (%):				
Mild	15 (43)	32 (70)	32 (89)	**<0.001**
Moderate	11 (31)	10 (22)	3 (8)	
Severe	9 (26)	4 (9)	1 (3)	
Extrapancreatic complications, n (%)	30 (75)	27 (52)	15 (38)	**0.003**
Infection, n (%)	15 (38)	6 (12)	3 (8)	**0.012**
Transfer to ICU, n (%)	5 (13)	2 (4)	1 (3)	0.119
In-hospital mortality, n (%)	4 (10)	2 (4)	1 (3)	0.272
LOH (days), mean ± SD	10 (10)	5 (7)	4 (3)	**0.002**

PMA: Psoas muscle area; SD: standard deviation; BMI: Body mass index; CRP: C-reactive protein; CT: Computed tomography; AP: Acute pancreatitis; ICU: Intensive care unit; LOH: Lenth of hospitalization. *Available for 88 patients.
